# Associated congenital genitourinary and intestinal anomalies: A case report

**DOI:** 10.1016/j.eucr.2025.103250

**Published:** 2025-10-21

**Authors:** Noemi Aparecida Betini Venturim, Laura de Paiva Rodrigues da Silva, Bárbara Eugênio Custódio Silva, Lyvia do Prado Pacheco, Lara Fachetti de Souza, Alexander Hatsumura Casini, Antônio Chambô Filho

**Affiliations:** a Department of Obstetrics and Gynecology, Hospital Santa Casa de Misericórdia de Vitória, Dr. João dos Santos Neves Street, 143, 29025-023, Vitória, Espírito Santo, Brazil; b Department of Urology, Hospital Santa Casa de Misericórdia de Vitória, Dr. João dos Santos Neves Street, 143, 29025-023, Vitória, Espírito Santo, Brazil

**Keywords:** Urogenital abnormalities, Didelphys uterus, Situs inversus

## Abstract

The female urogenital tract originates in the intermediate mesoderm, with the Müllerian ducts being critical for uterine, vaginal, and tubal formation. This report presents a 44-year-old patient with uterus didelphys, urethral and bladder duplication, complete longitudinal vaginal septum and intestinal malrotation, suggesting a complex, multi-stage embryonic disorder. The patient, asymptomatic from a gynecological viewpoint, presented with refractory urinary incontinence. Magnetic resonance imaging was crucial in characterizing the anatomy. The rarity and complexity of such cases underscore the need for a comprehensive classification system and multidisciplinary care to enable accurate diagnosis, appropriate management, and improved patient quality of life.

## Highlights

• A 44-year-old woman presented with a complex, multi-stage embryonic disorder.
• The patient had a rare condition involving genitourinary and intestinal anomalies.
• A comprehensive classification system and multidisciplinary care are required.

## 1 Introduction

To understand the functioning of the human body and its potential alterations, it is essential to clarify the tissue formation process and the eventual errors that could occur. The multiple cell types proliferate and undergo differentiation in a complex genetic and hormonal interaction, with the intermediate mesoderm being responsible for the development of the urogenital tract. A mutation or error occurring at any stage may give rise to duplicated, absent or malformed structures.^1^

According to Williams,^1^ the urogenital sinus is divided into three parts: (1) the cranial or vesicular portion, which gives rise to the urinary bladder; (2) the middle or pelvic portion, where the female urethra originates; and (3) the caudal or phallic portion, which gives rise to the distal vagina and the major vestibular glands (Bartholin's glands), urethral glands and paraurethral glands (Skene's glands). The normal development of the Müllerian ducts results in the uterus, Fallopian tubes and the upper portion of the vagina. Due to this close embryological relationship between the mesonephric (Wolffian) duct and the paramesonephric (Müllerian) duct, associated congenital anomalies involving the kidneys, ureters and the reproductive tract are common, with those involving the ovaries, skeleton, auditory tract and gastrointestinal tract being less common.^1^, ^2^, ^3^

Congenital malformations of the female reproductive system are classified according to the anatomical deviation from which they originated. Initially, the Müllerian ducts are divided by septa and, later, fusion occurs in a bidirectional craniocaudal manner to create a complete structure in the midline. A unicornuate uterus with no rudimentary horn is the result of a failure of the ducts to develop, generating aplasia in parts of the system. The unicornuate uterus with a rudimentary horn is caused by failure of the two Müllerian ducts to fuse. Uterus didelphys consists of two cervixes and two uteri, while the bicornuate uterus arises from the abnormal fusion or lack of fusion of the ducts. Finally, the septate uterus, the arcuate uterus and the obstructive or non-obstructive vaginal or cervical septum are the result of full or partial failure of the septum to absorb.^4^, ^5^, ^6^

Congenital anomalies of the urogenital tract are estimated to occur in 2–4 % of the female population, with up to 40 % of these cases being associated with renal malformations. A large part of the anomalies involving the renal pelvis and the ureter are due to duplication of the collecting system, a malformation that occurs when the ureteric bud divides at an early stage into two or more segments. This abnormal division hampers the normal differentiation of the upper urinary tract, resulting in relevant clinical anatomical variations. Episodes of recurrent urinary infections, urinary incontinence and urolithiasis may occur. The anomaly is more prevalent in women and is more often unilateral.^4^^,^^7^

Situs inversus abdominus, considered rare, is another congenital abnormality characterized by inversion of the abdominal organs in relation to their normal position. Individuals with this anomaly often remain asymptomatic throughout their lives or they may present with nonspecific abdominal pain, bowel obstruction, bowel ischemia, chronic and inexplicable abdominal discomfort, and even episodes of acute abdominal pain. Diagnosis is preferably made by computed tomography (CT), which shows the superior mesenteric vein situated on the left of the superior mesenteric artery instead of on the right in patients with malrotation.^8^

This case report describes a 44-year-old female patient with, concomitantly: uterus didelphys, vesical and urethral duplication, complete longitudinal vaginal septum and intestinal malrotation.

## 2 Case presentation

This report involved no intervention or monitoring of the patient. The data were retrospectively obtained from clinical, laboratory and imaging records following the patient's written consent. The patient was found to have uterus didelphys associated with bladder and urethral duplication, vaginal septum and intestinal malrotation while receiving care at the Department of Obstetrics and Gynecology of a referral hospital in Vitória, Espírito Santo, Brazil.

A 44-year-old hypertensive woman in use of enalapril presented in October 2024 for her first appointment at the urogynecology outpatient clinic complaining of urine leakage during physical exertion, while coughing and sneezing (stress incontinency); urinary urgency and nocturia. She refers that all symptoms began in her childhood, but worsened during adulthood, causing social impairment. The patient reported never having had sexual intercourse and informed having had surgery at six years of age due to fecal loss from the vagina (*recto-vaginal fistula?*). In addition, the patient reported having two uteri and two bladders.

**Fig. 1:**
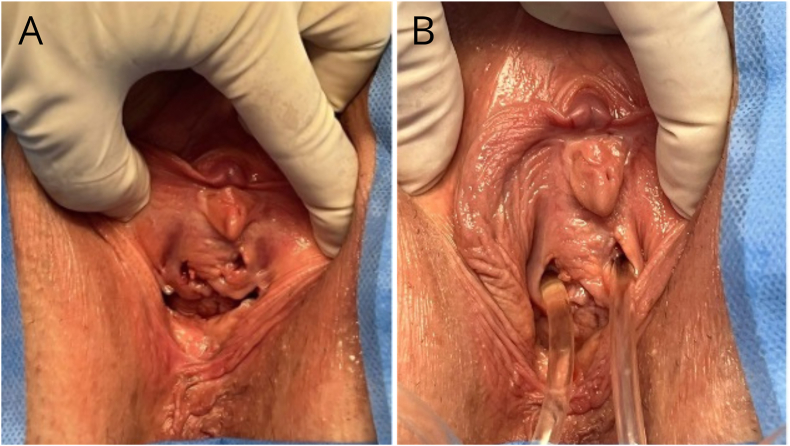
A- Outpatient physical examination with visualization of two urethras. B - Insertion of urinary catheter through the urethral ostia.

Physical examination confirmed the presence of two urethras (Fig. 1-A), which, when probed with a urinary catheter, were found to be patent, releasing clear urine (Fig. 1-B); complete longitudinal vaginal septum and, clearly visible, two vaginas. The hymen was intact. Since the patient had never had sexual intercourse, speculum and digital examinations were not performed. There was no urine loss following the Valsalva maneuver.

**Fig. 2:**
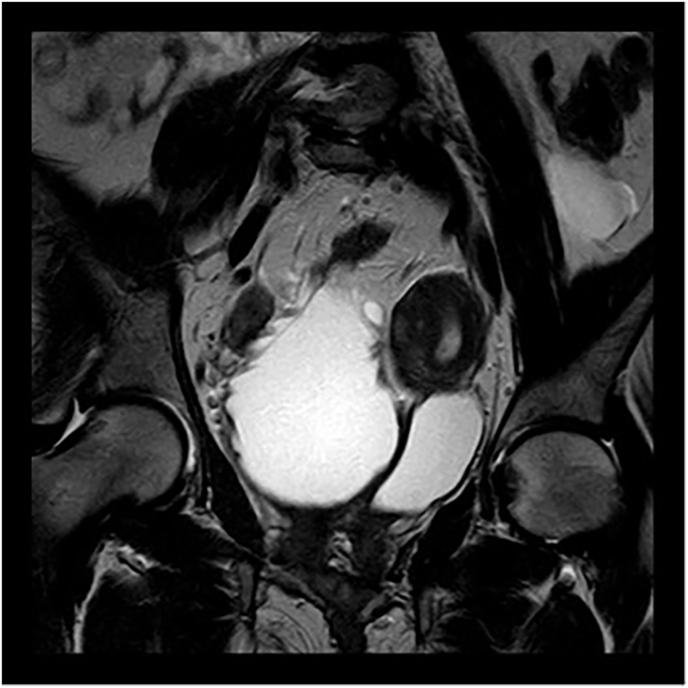
Magnetic resonance imaging, coronal plane. Duplicated bladder.

**Fig. 3:**
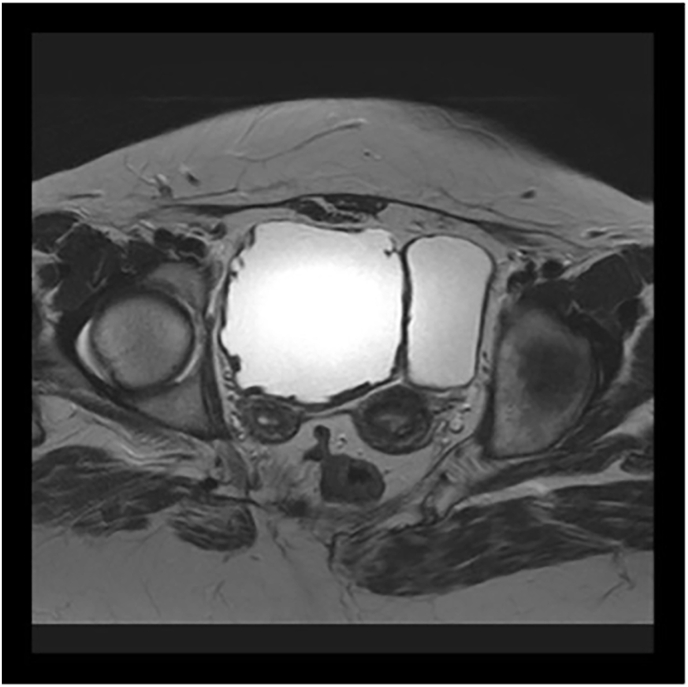
Magnetic resonance imaging, axial plane. Uterus didelphys and duplicated bladder.

Pelvic magnetic resonance imaging (MRI) in January 2023 showed complete duplication of the bladder in the sagittal axis, with two bladders positioned side by side communicating with the ipsilateral ureters without being connected to each other (Fig. 2, Fig. 3). Two urethras were also seen. The content of the bladders was homogenous, with the bladder on the right presenting diffuse parietal thickening, suggesting stress bladder. Regarding the uterus, there was complete separation of the corpuses and cervices, with, in addition, separation of the vagina (uterus didelphys and duplication of the vagina). Regular myometrial layers with normal signal intensity. Regular junctional zones with usual signal intensity. Endometrial and endocervical cavities with usual thickness.

**Fig. 4:**
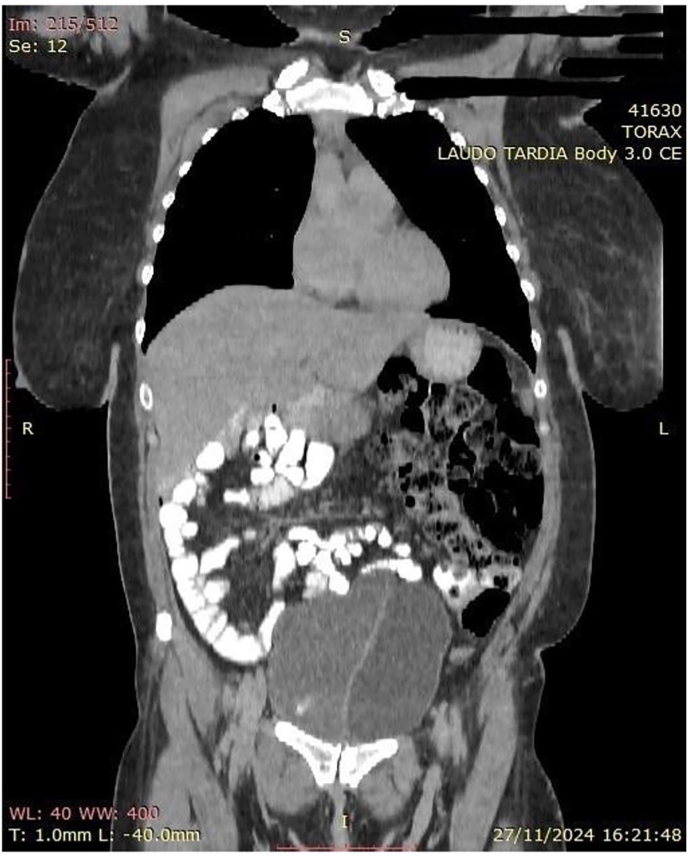
Computed tomography, coronal plane. Loops of the large intestine on the left; loops of the small intestine on the right; two bladders.

**Fig. 5:**
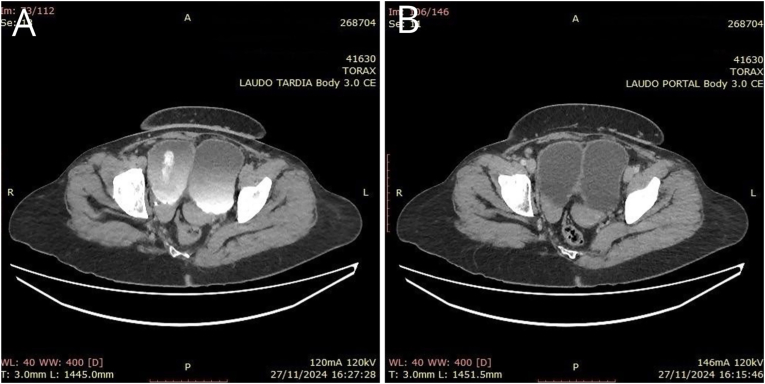
Computed tomography, axial plane: A - Uterus didelphys. B - Duplicated bladder.

In addition, an abdominal CT scan was performed in November 2024, due to suspicion of an associated digestive tract malformation, given that concomitant intestinal malformations are common in such cases. The CT showed complete duplication of the urinary bladder in the sagittal axis, with two bladders positioned side by side, and signs suggestive of stress urinary incontinence and/or an inflammatory or stress process. Uterus with complete separation of the uterine horns and possibly of the vagina (didelphys?). Rotation of the loops of the colon towards the left hemiabdomen and the loops of the small intestine to the right (Fig. 4, Fig. 5).

Oxybutynin was initiated as a therapeutic trial. Two weeks later, the patient returned to outpatient gynecology for further evaluation, still complaining of urinary urgency. At that time, the patient was also evaluated by the urology team, which, in conjunction with gynecology, decided to discontinue the medication, initiate pelvic physiotherapy, and perform a cystoscopy study for better anatomical assessment, including the investigation of potential obstructive factors.

One month later, the patient was admitted to the hospital for cystoscopy. The procedure was performed by the urology service with the patient in the lithotomy position. Two urethras were seen with the naked eye. Cystoscopy of the left urethral meatus showed: trabeculated bladder; single urethral meatus on the left. Cystoscopy of the right urethral meatus showed: trabeculated bladder, with some small diverticula, and abnormalities that contribute to stress urinary incontinence. Single urethral meatus on the right. The probe was then removed, and the bladder emptied. The patient was discharged from the hospital after receiving all necessary information about her health status, the confirmed diagnoses and the absence of a corrective surgical proposal. She was advised to continue clinical treatment guided by her symptoms, including pelvic physical therapy, and to undergo regular outpatient follow-up.

## 3 Discussion

This case report describes a patient with a Müllerian duct anomaly associated with renal and intestinal anomalies, specifically: uterus didelphys, complete longitudinal vaginal septum, two completely separate and functional bladders with two urethras and intestinal malrotation. It was evident that this was not merely a malfunction at one specific stage of embryonic development but, rather, one that involved various stages, resulting in this unique syndrome.

The formation of the female genital tract is complex and depends on a series of events involving cell differentiation, migration, fusion and canalization. Faults in any one of these processes results in a congenital anomaly. In the present case, a possible fault in lateral fusion was located, which could be a defect in reabsorption or in the fusion of the Müllerian ducts. This would lead to duplication of the reproductive structures, resulting in two separate uteri, each with its own cervix. This would also explain the longitudinal vaginal septum, present in 75 % of cases of uterus didelphys.^9^

The development of the reproductive tract is closely associated with that of the urinary system, both originating from urogenital ridges; therefore, genital tract anomalies are often associated with renal, ureteral or bladder anomalies. Congenital renal anomalies have been found in almost half of cases of Müllerian duct anomalies, with unilateral renal agenesis being the most common. Even with the established correlation between these anatomical anomalies, it is still evident that clinical practice lacks routine investigative protocols, resulting in delayed diagnosis.^3^

Intestinal malrotation is a congenital anomaly caused by incomplete or complete lack of rotation of the bowel along the axis of the superior mesenteric artery during embryo development. When symptomatic, up to 90 % of cases are diagnosed in the first year of life. When diagnosed in adulthood, the majority of individuals, however, are asymptomatic. Contrast tomography of the patient in question shows rotation of the loops of the colon towards the left hemiabdomen and the loops of the small intestine to the right; however, up to the present time, the patient has no bowel complaints.^10^

Classifying congenital abnormalities of the urinary and reproductive systems remains a challenge, as discussed by Grimbizis and Campo.^5^ According to the most recent American Society for Reproductive Medicine (ASRM) classification, which is based on the anatomy of the female genital tract, this patient would be considered class III (uterus didelphys). The classification proposed by Acien et al., in 2004, however, takes clinical and embryological factors into consideration, with category 5 being applicable to this case (a combination of malformations: Wolffian duct, Müllerian duct and cloacal anomalies). The VCUAM (Vaginal, Cervix, Uterus, Adnexa and associated Malformations) classification system is based on anatomy; however, due to its lack of practicality, it is not routinely used. Nevertheless, the present case would be classified as: V2b,C1,U+,AO,MRM+: complete septate vagina (V2b); duplex cervix (C1); Uterus – other (didelphys) (U+); normal adnexa (A0); associated malformations – renal and other (bowel) (MR, M+).

These classifications are currently considered obsolete due to the fragilities of the classification system and its applicability as routine. The need for an innovative classification system that would encompass the positive points of each existing system in a practical way is clear. The relevant gaps concern clinical, prognostic and therapeutic correlations as well as the broad-reaching incorporation of all the potential variables, since, with the increasing precision of diagnostic methods, previously undocumented anatomic syndromes are now being identified.^5^^,^^9^

Clinically, the condition is generally nonspecific; however, pelvic pain, abnormal bleeding at menarche, recurrent miscarriage or premature delivery are often reported. Nevertheless, the patient here is gynecologically asymptomatic, with her principal complaint being urinary incontinence. Cystoscopy revealed signs of stress urinary incontinence; however, oxybutynin was ineffectual. Regarding diagnostic methods, ultrasonography was important, while MRI was crucial in providing clear and precise details of the female genital tract. These imaging tests, however, are mostly requested for other reasons, with the diagnosis of congenital abnormalities often being incidental.^11^

Surgical treatments aimed at restoring the anatomy to normal are reserved for symptomatic patients without confirmed primary infertility. Approaches include, for example, hysteroscopic metroplasty on the septate uterus and reunification of the uterus via laparotomy (Strassman metroplasty) in cases of bicornuate uterus.^12^^,^^13^

## 4 Conclusion

The present report highlights the complexity of congenital anomalies of the genitourinary and gastrointestinal tracts. The case described here reports on a rare association of uterus didelphys, bladder and urethral duplication, vaginal septum and intestinal malrotation, reinforcing the importance of a profound understanding of embryology for the adequate diagnosis and management of these conditions.

The simultaneous involvement of the reproductive and urinary systems, originating from the urogenital ridges, explains the frequent occurrence of anomalies in these systems, making a detailed investigation of both systems crucial in suspected cases.

A multidisciplinary approach is essential in guaranteeing comprehensive and individualized care, improving clinical management and the quality of life of patients with complex congenital malformations.

## CRediT authorship contribution statement

**Noemi Aparecida Betini Venturim:** Writing – original draft, Visualization, Project administration, Methodology, Investigation, Data curation, Conceptualization. **Laura de Paiva Rodrigues da Silva:** Writing – review & editing, Writing – original draft, Visualization, Project administration, Investigation, Conceptualization. **Bárbara Eugênio Custódio Silva:** Investigation, Data curation, Conceptualization. **Lyvia do Prado Pacheco:** Writing – original draft, Project administration, Methodology, Investigation, Data curation, Conceptualization. **Lara Fachetti de Souza:** Visualization, Investigation, Data curation, Conceptualization. **Alexander Hatsumura Casini:** Validation, Supervision, Resources, Investigation. **Antônio Chambô Filho:** Writing – review & editing, Validation, Supervision, Resources, Investigation.

## Ethics statement

This study was conducted in accordance with the Brazilian National Health Council's recommendations as laid out in Resolution 466 of 2012 and was approved by the institution's internal review board.

## Consent for publication

The patient was provided with information and agreed to the publication of this case report by signing an informed consent form. Confidentiality was guaranteed.

## Data statement:

De-identified data may be shared upon reasonable request to the corresponding author.

## Funding

This research did not receive any specific grant from funding agencies in the public, commercial, or not-for-profit sectors.

## Conflicts of interest

None.
